# *Salvia miltiorrhiza* Roots against Cardiovascular Disease: Consideration of Herb-Drug Interactions

**DOI:** 10.1155/2017/9868694

**Published:** 2017-04-03

**Authors:** Feng Chen, Li Li, Dan-Dan Tian

**Affiliations:** ^1^Hainan Provincial Key Laboratory of R&D of Tropical Herbs, School of Pharmacy, Hainan Medical College, Haikou 571199, China; ^2^State Key Laboratory of Drug Research, Shanghai Institute of Materia Medica, Chinese Academy of Sciences, Shanghai 201203, China; ^3^Department of Pharmaceutical Sciences, College of Pharmacy, Washington State University, Spokane, WA 99212, USA

## Abstract

*Salvia miltiorrhiza* root (Danshen) is widely used in Asia for its cardiovascular benefits and contains both hydrophilic phenolic acids and lipophilic tanshinones, which are believed to be responsible for its therapeutic efficacy. This review summarized the effects of these bioactive components from* S. miltiorrhiza* roots on pharmacokinetics of comedicated drugs with mechanic insights regarding alterations of protein binding, enzyme activity, and transporter activity based on the published data stemming from both* in vitro* and* in vivo* human studies.* In vitro* studies indicated that cytochrome P450 (CYP450), carboxylesterase enzyme, catechol-*O*-methyltransferase, organic anion transporter 1 (OAT1) and OAT3, and P-glycoprotein were the major targets involved in* S. miltiorrhiza*-drug interactions. Lipophilic tanshinones had much more potent inhibitory effects towards CYPs activities compared to hydrophilic phenolic acids, evidenced by much lower *K*_*i*_ values of the former. Clinical* S. miltiorrhiza*-drug interaction studies were mainly conducted using CYP1A2 and CYP3A4 probe substrates. In addition, the effects of coexisting components on the pharmacokinetic behaviors of those noted bioactive compounds were also included herein.

## 1. Introduction

Cardiovascular disease, one of the top leading causes of mortality across the world, is responsible for 31% of deaths in 2012 [1]. Frequently prescribed medications include anticoagulants, calcium-channel blockers, *β*-blockers, diuretics, and platelet aggregation inhibitors. Moreover, cardiovascular disease is often accompanied by obesity and/or diabetes. Polypharmacology, which describes multitargets with multiple-medications exemplified by herbs, has suggestive superior efficacy and safety and is less liable to adverse effects compared to single-medication [2]. Herbal medicines usually have a variety of constituents exerting polypharmacological roles against multiple targets. As high as 80% of the population in Africa and Asia [3] and 4–61% cardiac patients [4] take traditional medicines for health benefits. Patients seek herbs for boosting pharmacologic effects, relieving unwanted adverse syndromes caused by western drugs, and reducing economic burden [5, 6].* Salvia miltiorrhiza* root (Danshen) is widely used, alone or coprescribed with other herbs or conventional drugs, by patients with angina pectoris and myocardial infarction in Asia [7, 8]. A meta-analysis of sixty randomized clinical trials indicated that Danshen Dripping pill, consisting of* S. miltiorrhiza*,* Panax notoginseng*, and* Dryobalanops camphora*, showed much more apparent efficacy compared to isosorbide dinitrate [9].

The combination of cardiovascular drugs and* S. miltiorrhiza* roots highlights potential interactions caused by pharmacokinetic and pharmacodynamic mechanisms. Pharmacokinetic interaction often leads to increased or decreased victim drug systemic levels due to alternation of absorption, distribution, metabolism, and excretion by perpetrator drugs [10, 11]. Underlying mechanisms include but are not limit to protein binding and inhibition and induction of enzyme/transporter activities [12]. These interactions at the end may cause attenuated efficacy or unwanted toxicity, which suggests necessary dose adjustment.

**Figure 1:**
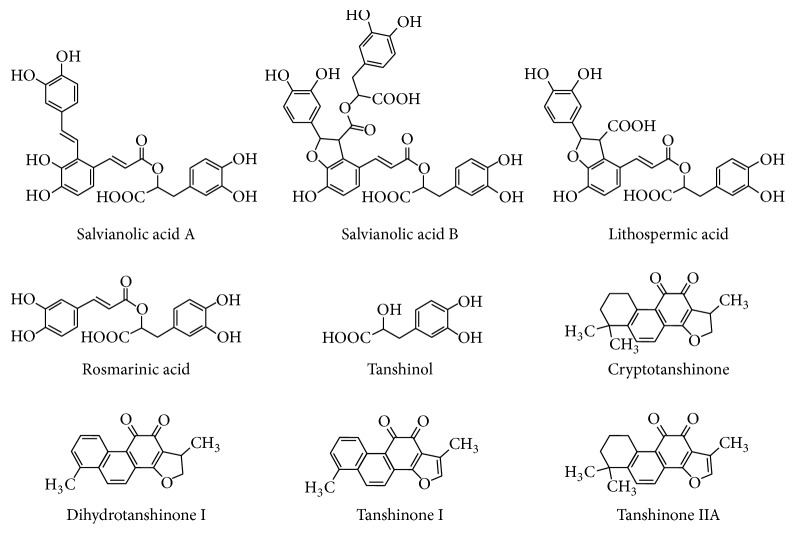
Structures of major constituents from Danshen.

Around one hundred components are found in* S. miltiorrhiza *roots [13], which are classified into two groups: (1) hydrophilic phenolic acids with tanshinol, rosmarinic acid, lithospermic acid, and salvianolic acids A and B as the major ones; (2) lipophilic tanshinones with tanshinone I, tanshinone IIA, cryptotanshinone, and dihydrotanshinone I as the major ones (Figure 1). The contents of chemicals in* S. miltiorrhiza* roots vary among aqueous extracts, organic solvent extracts, oral pills, and injections derived from the single herb of* S. miltiorrhiza* roots or herbal combination, like Fufang prescription in China. These compounds can cause coronary vasodilatation, suppress thromboxane formation, inhibit platelet adhesion and aggregation, and scavenge free radicals [13]. Therefore, roles of* S. miltiorrhiza* either as a perpetrator or as a victim need to be explored in herb-drug interactions.

In this review, we mainly focus on pharmacokinetic interaction between* S. miltiorrhiza* roots and drugs both in human* in vitro* studies and in clinical trials. Animal* in vivo* studies are also included when clinical studies are not available. In addition, influence on pharmacokinetics of bioactive constituents from* S. miltiorrhiza* roots by other combined herbs or drugs is also explored herein.

## 2. Pharmacokinetic Interaction Caused by* Salvia miltiorrhiza*

### 2.1. Protein Binding

After administration, a drug circulates in bloodstream as both free form and protein-drug complex. Acidic and neutral drugs primarily bind to alkalotic albumin and basic drugs tend to bind to *α*1-acid glycoprotein and lipoproteins [14, 15]. Based on “free drug hypothesis,” free drug concentrations are deemed critical for efficacy/safety since only free drugs can penetrate membrane barriers and have the best correlation with drug response [16]. Pharmacological effect of drugs can be changed when unbound concentration changes while total concentration remains the same [17, 18]. Displacement may occur and lead to elevated unbound fractions in the presence of another drug.

Human serum albumin is the most abundant protein in circulation and bovine serum albumin has a similar structural homology. These two albumins are commonly used for evaluating drugs binding affinities* in vitro*. Drugs binding constants, the higher values of which indicate more tight binding, determine their unbound concentrations in blood. Salvianolic acid B and rosmarinic acid can bind to bovine serum albumin at site I with binding constants around 10^5^ L/mol [19]. Salvianolic acid B, lithospermic acid, rosmarinic acid, salvianolic acid A, and salvianolic acid C can bind to human serum albumin at sites I and/or II with increasing binding constants at 298 K ranging from 0.18 to 16 × 10^5^ L/mol [20–22]. The binding constant for tanshinone IIA at 303 K was 1.54 × 10^5^ L/mol [23]. The plasma protein binding of salvianolic acid A, salvianolic acid B, and tanshinone IIA was 99.7% [24], 83.8–92.1% [25, 26], and 99.2% [27] while that of tanshinol was 2% [7]. Warfarin, an anticoagulant preventing formation of blood clots, is highly bound (97–99%) with human serum albumin in plasma with selectivity to site I. In the presence of the above-mentioned individual constituent, the human serum albumin binding constant of warfarin decreased by 1.4- to 8.7-fold [20] which may cause increased free warfarin concentrations. Sodium tanshinone IIA sulfonate, a water-soluble derivative of tanshinone IIA used as an injection in China, displaced warfarin from the warfarin-human serum albumin complex, the signal of which can be directly measured by mass spectrum [28]. Therefore, increased levels of free plasma warfarin could be observed when warfarin is combined with Danshen because of replacement of warfarin by its constituents, which may potentiate anticoagulant response of warfarin.

Using an ex vivo study, the effect of coadministration of Danshen-Gegen (Radix* Puerariae lobatae*) formula on warfarin protein binding was investigated. The rats were orally given the formula at a single dose of 0.15 g/kg or at 0.15 g/kg twice daily for five days. Blood was collected 30 min and 2 h after the single dosing and the last multiple dosing, respectively. By adding warfarin to the freshly obtained plasma, a 12–22% increase of unbound warfarin concentrations was observed. Due to the highly bound fraction of warfarin in rat plasma (92%), the authors recommended that this change of unbound warfarin levels cannot be overlooked and concomitant use of Danshen-Gegen and warfarin for treating cardiovascular disease should be avoided [29].

In addition to warfarin, there are other drugs that can be affected by displacement of protein binding by constituents from Danshen. Ethyl acetate extract of Danshen, 50–70% of which bound to albumin, can displace salicylate in serum resulting in as high as 1.6-fold increase in free salicylate concentration [30]. Lipophilic constituents were responsible for the effect since they were more easily extracted by ethyl acetate than hydrophilic phenolic acids occurring in Danshen.

### 2.2. Metabolic Enzyme Activity

Most drugs undergo metabolism by enzymes in different tissues after they enter the body and produce metabolites with or without pharmacological activities. Regulation of enzyme activities can alter the systemic exposure and eventually the efficacy of a drug. Cytochromes P450 (CYP450) enzyme-mediated drug interaction is one of the most popular research topics due to well-known specific inhibitors and probe substrates for CYP isozymes.

Using human liver microsomes (HLMs) and recombinant CYP isozymes, different inhibitory potency against various CYPs have been observed for Danshen extract and its individual constituents [7, 31–39] (Table 1). Water-soluble constituents from Danshen tend to exert little or weak inhibitory effect on CYPs activities except for tanshinol and salvianolic acid B. Tanshinol inhibited CYP1A2, CYP2C8, CYP2C9, and CYP2C19 activities with IC50 of 110, 34, 99, and 67 *μ*M, respectively [7, 37]. Salvianolic acid B inhibited CYP1A2 with IC50 of 105 *μ*M and protocatechuic aldehyde suppressed CYP3A4 activity with IC50 of 130–160 *μ*M [37]. An IC50 value of 51 *μ*g/mL against CYP3A4 was found when testing the inhibitory roles of a Danshen extract, in which the content of tanshinones was approximately two orders of magnitude less than that of water soluble phenolic acids [35]. Salvianolate, a highly purified aqueous extract from Danshen, consists of salvianolic acid B (≥85%), rosmarinic acid (≥10.1%), and lithospermic acid (≥1.9%). Salvianolate can inhibit CYP3A4 using HLMs with *K*_*i*_ value of 2.27 *μ*g/mL and using recombinant CYP3A4 with IC50 value of 3.58 *μ*g/mL, whereas the other CYPs were hardly affected by this extract indicated by the IC50 values higher than 100 *μ*g/mL [32]. Using HepG2 cells, salvianolic acid B at 1 and 10 *μ*M downregulated CYP3A4 mRNA expression with or without induction by rifampicin. Meantime, decrease of CYP1A2 mRNA expression was also observed at the same concentration of salvianolic acid B [40]. However, CYP3A4 and CYP1A2 protein levels and activities were not evaluated.

Compared to hydrophilic constituents, lipophilic tanshinones tend to have much more potent inhibitory effects towards CYPs activities. Tanshinone I, tanshinone IIA, cryptotanshinone, and dihydrotanshinone I inhibited CYP1A2 with IC50 values ranging from 0.02 to 3.0 *μ*M using either HLMs or recombinant CYP1A2 [36, 39, 41]. Similarly, tanshinone I, cryptotanshinone, and dihydrotanshinone I inhibit CYP2E1 with IC50 values ranging from 0.7 to 10 *μ*M. Cryptotanshinone and dihydrotanshinone I inhibit CYP2C9 with IC50 values ranging from 7.5 to 33 *μ*M. Tanshinone I, tanshinone IIA, and cryptotanshinone were weak inhibitors (IC50 > 75 *μ*M) towards CYP2D6 and CYP3A4 [41]. However, dihydrotanshinone I exerted a potent inhibitory role against CYP3A4 with IC50 at 1.2–3.2 *μ*M [33, 36]. Because of more potent inhibitory effect of tanshinones than phenolic acids, ethanol extract which usually contained mainly tanshinones exerted much lower IC50 and *K*_*i*_ values compared to aqueous extract. It was reported that ethanol extract could inhibit CYP1A2 and CYP3A4 in HLMs with IC50 or *K*_*i*_ values of 3.4 and 8.6–12 *μ*g/mL, respectively [32, 33]. Sodium tanshinone IIA inhibited CYP3A4 with *K*_*i*_ value of 3.2 *μ*M in HLMs, whereas it had little inhibitory effect on CYP1A2, CYP2A6, CYP2C9, CYP2C19, CYP2D6, and CYP2E1, supported by much higher IC50 values (>100 *μ*M) [31].

Besides inhibition, Danshen ethanol extract, tanshinone IIA, and cryptotanshinone could induce CYP3A4 via activating human pregnane X receptor and, to a less extent, constitutive androstane receptor and glucocorticoid receptor in HepG2 cells [42]. Tanshinone I, tanshinone IIA, cryptotanshinone, and dihydrotanshinone I were reported to induce CYP1A1/2 via aryl hydrocarbon receptor in the human HepG2 cells and lead to 2- to 14-fold increase of CYP1A1/2 activity with concentration of individual component at 5 *μ*M [43].

In addition, UDP-glucuronosyltransferases (UGTs) as well as CYP3A4 are targets of pregnane X receptor. Therefore, it is possible that Danshen could induce UGTs expression levels [33]. Meanwhile, salvianolic acid B at 1 and 10 *μ*M increased glutathione S-transferase protein levels in HepG2 cells [40]. Using* o*-nitrophenyl acetate and irinotecan as substrates of human carboxylesterase (CE) enzyme 2, miltirone and cryptotanshinone had the most potent inhibition demonstrated with *K*_*i*_ values of 0.04 and 0.08 *μ*M, 0.14 and 0.29 *μ*M, respectively. Tanshinone IIA and tanshinone I only exhibited potent inhibitory effect towards irinotecan metabolism with *K*_*i*_ value of 0.07 and 0.14 *μ*M, respectively, and dihydrotanshinone I only inhibited* o*-nitrophenyl acetate metabolism with *K*_*i*_ value of 0.12 *μ*M. Unlike CE2, the inhibitory effects of tanshinones against CE1 were modest with cryptotanshinone and dihydrotanshinone as exceptions, evidenced by *K*_*i*_ values of 0.54 and 0.40 *μ*M, respectively [44]. Compared to CYP enzymes, more studies are necessary for investigating the effect of Danshen on non-CYP enzymes.

Collectively, hydrophilic phenolic acids and lipophilic tanshinones have exerted different inhibitory potency for various CYPs, the latter of which are more likely to cause Danshen-drug interactions. Among hydrophilic constituents from Danshen, tanshinol had the highest bioavailability (40% in dogs and 30% in rats) while other phenolic acids were slightly or not detected in human and dogs after oral administration [7, 45]. The oral bioavailability of salvianolic acid B and lithospermic acid in rats was only 0.02% [46] and 1.15% [47]. After oral administration of cardiotonic pills containing 4.8 mg of tanshinol, the maximum plasma concentration (*C*_max_) of tanshinol was only 20–30 ng/mL [45]. Meanwhile, tanshinones tend to have *C*_max_ in the nanomolar to lower micromolar range [48] due to their low contents in commonly used aqueous Danshen extract and poor bioavailability. For example, the oral bioavailability of cryptotanshinone was 2.1% in rats [49]. Therefore, there is minor possibility that traditional oral medication of aqueous Danshen extract-derived products can cause CYP-mediated Danshen-drug interaction. However, it is probable for Danshen-derived injections or other formulations with high content tanshinones to lead to Danshen-drug interactions. Furthermore, all the reported data on enzyme inhibition and induction by Danshen were based on liver. High exposure of intestinal enzymes to herbal constituents in the intestine is also a critical factor contributing to herb-drug interactions [50]. Therefore, further investigations concerning gut enzyme modulation by Danshen are necessary in the future.

### 2.3. Transporter Activity

Uptake and efflux transporters, as well as enzymes, are critical determinants of drug systemic exposures and efficacies. Modulation of transporters functions can lead to altered drug pharmacological effects [51–53]. Lithospermic acid, rosmarinic acid, salvianolic acid A, salvianolic acid B, and protocatechuic acid were reported to have inhibitory effects towards human organic anion transporter 1 (OAT1) and OAT3 with *K*_*i*_ values of 20.8 and 0.59, 0.35 and 0.55, 5.6 and 0.16, 22.2 and 19.8, and 13.4 and 81.8 *μ*M, respectively [54, 55]. Tanshinol exhibited weak inhibitory potency towards human OAT1, OAT2, OAT3, and OAT4 with IC50 values of 98, 1528, 2803, and 4079 *μ*M, respectively, using transfected HEK293 cells. However, tanshinol at 1 mM did not inhibit human organic anion transporting polypeptide 4C1 (OATP 4C1), organic cation transporter 2 (OCT2), organic cation/carnitine transporter 1 (OCTN1), multidrug and toxin extrusion protein 1 (MATE1), and MATE2-K in transfected HEK293 cells or P-glycoprotein (P-gp), multidrug resistance-associated protein 2 (MRP2), MRP4, and breast cancer resistance protein (BCRP) in inside-out membrane vesicles that expressed specific transporter [7, 56]. In addition, treatment with salvianolic acid A at 3, 6, and 12 *μ*M for 48 h in MCF-7/PTX cells significantly downregulated both gene and protein expression of P-gp, MRP1, and BCRP [57]. After intravenous administration of a Danshen injection to healthy subjects, the unbound *C*_max_ for rosmarinic acid, salvianolic acid B, and tanshinol were 0.53, 0.39, and 18 *μ*M, respectively [54, 58]. Considering unbound plasma concentration/*K*_*i*_ (or IC50) ratios caused by potent inhibitory effect of rosmarinic acid and high plasma concentration of tanshinol after administration of Danshen injections, OAT1 and OAT3 mediated Danshen-drug interactions possibly take place during the clinical practice.

Cryptotanshinone and dihydrotanshinone I inhibited P-gp indicated by decreased efflux ratio of digoxin (a cardiac glycoside) transport across Caco-2 monolayer in the presence of these two components compared to baseline. The inhibitory effect was due to downregulated P-gp mRNA and protein expression. P-gp protein levels in P-gp overexpressing SW620 Ad300 cells decreased by 40% after incubation with 12.5 *μ*M of individual cryptotanshinone and dihydrotanshinone I for 24 h. Miltirone failed to inhibit the P-gp mediated transport of digoxin in Caco-2 cells [59]. Reports on effects of tanshinone I and tanshinone IIA towards P-gp function were controversial. Hu et al. found little inhibitory effect [59], whereas Li et al. [60] and Yu et al. [61] reported significant inhibitory effects of tanshinone I (IC50 = 0.53 *μ*M) and tanshinone IIA (IC50 = 2.6 *μ*M) on P-gp activity, respectively.

Since Danshen ethanol extract, tanshinone IIA, and cryptotanshinone could activate human pregnane X receptor [42], via which P-gp can be induced, it is hypothesized that Danshen lipophilic constituents might induce P-gp. Indeed, treatment of tanshinone IIA and cryptotanshinone at 10 *μ*M for 72 h exerted comparable inductive effects on P-gp mRNA with rifampin at 25 *μ*M using primary human hepatocytes [62]. Upregulation of mRNA levels of BCRP, human peptide transporter 1 (hPepT1), monocarboxylate transporter (MCT), and MRP1-6 in Caco-2 cells were also found after treatment by tanshinone IIA at 0.01 or 1 *μ*M for 24 h [63]. The low bioavailability of tanshinones restrained them from entering the liver at high concentrations. Therefore, it is probable that tanshinones might cause P-gp mediated interactions with a much greater extent in the gut than in the liver after oral administration of Danshen products.

### 2.4. Clinical Studies

Clinical studies on pharmacokinetic Danshen-drug interactions are listed in Table 2. Two-week treatment with Danshen extract tablets had no effect on the pharmacokinetics of theophylline that metabolized predominantly by CYP1A2 and, to a less extent, CYP2E1 [65], which was due to low bioavailability of tanshinones and weak inhibitory potency of hydrophilic constituents [37]. Following this study, Qiu et al. reported [67] that two-week treatment of the same Danshen extract tablets caused 31% and 27% decrease of midazolam *C*_max_ and AUC_0–*∞*_, respectively, in volunteers. It has been confirmed that tanshinones had inductive effects on CYP1A, CYP2C, and CYP3A in mice while aqueous extract of Danshen did not [69]. Therefore, the decreased midazolam levels may be due to both hepatic and intestinal CYP3A4 induction by lipophilic constituents [33, 42]. Ten-day treatment of Danshen ethanol extract, which contained 26- to 85-fold higher tanshinones compared to the above mentioned Danshen extract tablets, decreased the midazolam AUC_0–12 h_ and *C*_max_ values by 80% and 66%, respectively, accompanied by a 3.0-fold increase of the AUC ratio of 1-hydroxymidazolam/midazolam [33]. Inductive effect of Danshen towards CYP3A4 was dependent on tanshinones dose although not proportionally, which was indicated by the extent of pharmacokinetic change of midazolam. As discussed earlier, tanshinones could induce P-gp* in vitro*. Importantly, consistent findings were observed in humans. Using the p-gp substrate fexofenadine as a probe, ethanol extract of Danshen for ten days can significantly decrease fexofenadine AUC_0–24 h_ and *C*_max_ by 45% and 35%, respectively [62].

Injection of sodium tanshinone IIA sulfonate at 60 mg/day for 13 days increased CYP1A2 activity (ratio of paraxanthine to caffeine at 6 h in plasma) by 41% and decreased caffeine AUC by 13%, suggesting induction of CYP1A2 activity [66]. *C*_max_ of sodium tanshinone IIA sulfonate in human was around 2 *μ*M after one-hour intravenous infusion at the dose of 40 mg [70]. Considering *K*_*i*_ value of 3.2 *μ*M towards hepatic CYP3A4 inhibition [31], interactions of tanshinone IIA sulfonate and CYP3A4 substrate drug would take place if they are administered simultaneously.

Danshen is often in combined use with other herbs in China. T89 consists of Danshen, Sanqi (Radix Notoginseng), and Borneol. The main components from Danshen in T89 are hydrophilic phenolic acids. After warfarin, which was metabolized primarily by CYP2C9, CYP1A2, and CYP2C19 [71], reached steady-state in healthy volunteers, T89 was added in combination with warfarin for another week. T89 had no significant effect on total concentration and pharmacodynamic effect of warfarin [68]. This nonsignificant change in warfarin pharmacokinetics is consistent with the* in vitro* finding that hydrophilic phenolic acids exhibited high IC50 values against CYP450 activities (Table 1), which cannot be reached after regular dose of Danshen. However, Danshen was reported to cause potentiation of anticoagulation of warfarin in patients in three case reports [72–74] and a clinical study [75]. The difference of effect of Danshen on warfarin pharmacodynamic effect among these studies may be due to differences of dosage of Danshen and heath conditions. Since no warfarin levels were detected in the above-mentioned patients, the existence of pharmacokinetic interaction between Danshen and warfarin was unclear and the underlying mechanisms still need to be explored.

Collectively, clinical studies mainly focused on influence of Danshen on substrate drugs of CYP450 or P-gp due to extensively published data regarding potent modulatory effects of its ingredients. After oral administration of Danshen products enriched in tanshinones, CYP1A2, CYP3A4, and P-gp mediated Danshen-drug interactions were observed. Clinical interactions of Danshen and cardiovascular drugs focused on Danshen-warfarin interactions with unclear mechanisms.

## 3. Pharmacokinetic Alterations of Danshen Bioactive Constituents due to Coexisting Components

Danshen is prescribed for cardiovascular protection either alone or in combination with other herbs such as Fufang Danshen in China. Since Danshen itself is a cardiovascular protective herb, the pharmacokinetics of its bioactive constituents, which can be affected by coexisting components from Danshen extract, other herbs, and drugs, is important for evaluating the efficacy and toxicity. Recently, studies in this field are mainly conducted using animal models.

When Danshen injection was coadministered to rats with pure tanshinol, protocatechuic aldehyde, salvianolic acid A, or salvianolic acid B at 10-fold of their corresponding levels in Danshen injection, a 27–83% increase in the AUC of tanshinol, salvianolic acid A, and salvianolic acid B was observed compared to control group due to pharmacokinetic interactions [76]. Intravenous injection of tanshinones and polyphenolic extracts caused 2- to 14-fold change in the AUC of tanshinone IIA and salvianolic acid B compared to dosing either tanshinones or polyphenolic extract alone [77]. Metabolism-based interactions could be the underlying mechanism. Tanshinones are a class of compounds that has low bioavailability due to poor absorption and P-gp mediated intestinal efflux [49, 60, 61]. Similarly to intravenous injection, oral administration of tanshinones extract could result in 4- to 18-fold increase in the systemic exposure of cryptotanshinone and tanshinone II A compared to that of oral administration of pure cryptotanshinone or tanshinone II A [78]. This might be partly due to the inhibition of p-gp mediated efflux during absorption. It was reported that coexisting components from Danshen enhanced intestinal absorption of cryptotanshinone by 30–40% via inhibiting efflux transport of cryptotanshinone by P-gp using a rat gut sac model [79].

The coadministration of Danshen and Sanqi significantly improved intestinal absorption (2-fold) of the bioactive component salvianolic acid B in rats compared to administration of Danshen alone, suggesting synergistic effect of these two herbs [80]. However, another rat study reported oral administration of Fufang Danshen preparation or salvianolic acid B extract alone or in the presence of Sanqi, Borneol, and tanshinones extract had the same salvianolic acid B pharmacokinetics. Similar results were found for tanshinone IIA [81]. Using Guinea pigs as the animal model, the values of *C*_max_ and AUC of salvianolic acid B in plasma decreased by 35% and 37%, respectively, and the corresponding values of tanshinone IIA decreased by 91% and 84%, respectively, after intratympanic administration of Danshen and Sanqi. Interestingly, 5.7- and 7.7-fold increase in AUC of salvianolic acid B and tanshinone IIA were found in cerebrospinal fluid [82]. This effect might be due to a different dosing route applied in guinea pigs.

To improve the absorption of tanshinol and salvianolic acid B, sodium caprate was dosed with Danshen tablet. *C*_max_ and AUC values of tanshinol increased by 1.8-fold and the AUC value of salvianolic acid B increased by 1.4-fold due to improved intestinal permeability with addition of sodium caprate [83]. In addition, a single dose of rifampin caused 2.6- to 7.0-fold and 1.9- to 2.7-fold increase in salvianolic acid B AUC and *C*_max_ values compared to baseline [84], respectively, the reason of which is partly due to inhibition of OATP-mediated elimination. Tanshinol was metabolized by methylation and, to a less extent, by sulfation into inactive metabolites. The metabolites and parent tanshinol were then subjected to OATs-mediated renal elimination both in human and in rat model [85]. Impairment of tanshinol methylation by entacapone, a potent catechol-*O*-methyltransferase inhibitor, caused 35% and 43% increase of tanshinol AUC with oral and intravenous administration of tanshinol to rats, respectively [7]. Blockage of tanshinol renal excretion by probenecid, an inhibitor of multiple OATs, led to 3.3-fold increase of tanshinol AUC in rats after intravenous administration of tanshinol [56]. Since tanshinol had a wide therapeutic effect window, the presence of catechol-*O*-methyltransferase and OATs inhibitors, from either herbs or comedicated drugs, might improve Danshen efficacy in cardiovascular treatment without causing unwanted adverse effects.

## 4. Conclusions

This review summarized pharmacokinetic Danshen-drug interactions* in vitro* and* in vivo*.* In vitro* studies indicated that lipophilic tanshinones tend to have more potent modulatory effects on enzyme and transporter activities than hydrophilic phenolic acids. Whether phenolic acids or tanshinones would cause significant* in vivo* pharmacokinetic Danshen-drug interactions depends not only on their intrinsic potency but also on their dosages. Different extraction methods and administration routs for Danshen products make the dosages of major bioactive components vary largely and cause difficulties in comparing those published research data among different labs. Information on dosages and pharmacokinetics of the major bioactive components from Danshen would help understand* in vivo* herb-interaction results. Therefore, it is highly recommended that researchers provide extraction details, contents of constituents in dosing formulations, and pharmacokinetics of major herbal components in the future research. Additionally, effects of coexisting components on pharmacokinetics of bioactive ingredients from Danshen were investigated mainly using animal models nowadays and further clinical studies are necessary for better disclosing underlying mechanisms of the therapeutic effect of this herb.

## Figures and Tables

**Table 1:** Inhibitory effects of Danshen extract and its bioactive constituents towards enzyme and transporter activities.

Extract/constituent	Enzyme/transporter	Substrate	*K*_*i*_ (*μ*M)			Reference
			HLMs	Purified	Inhibition type	
Tanshinone I	CYP1A1	7-Ethoxyresorufin		0.75^*∗*^		[39]
	CYP1A2	7-Ethoxyresorufin		0.19^*∗*^		[39]
	CYP1A2	Phenacetin	0.48–2.16	0.11	com	[36, 37]
	CYP2C9	Tolbutamide	51.2	73.4	com	[36]
		Diclofenac	>200^*∗*^			[37]
	CYP2D6	Dextromethorphan	120^*∗*^			[37]
	CYP2E1	Chlorzoxazone	3.67	ND	non	[36]
	CYP3A4	Testosterone	86.9	92.5	com	[36]
		Testosterone	>200^*∗*^			[37]
		Midazolam	>200^*∗*^			[37]
	CE1	*o*-Nitrophenyl acetate		26.3		[44]
	CE2	*o*-Nitrophenyl acetate		14.6		[44]
		Irinotecan		1.4		[44]
	P-gp	Digoxin	0.53^*∗*^ (Caco-2 cells)			[60]

Tanshinone IIA	CYP1A	7-Ethoxyresorufin	0.2^*∗*^			[39]
	CYP1A	7-Methoxyresorufin	0.38^*∗*^			[39]
	CYP1A1	7-Ethoxyresorufin		4.3^*∗*^		[39]
	CYP1A2	7-Ethoxyresorufin		0.09^*∗*^		[39]
	CYP1A2	Phenacetin	1.0–1.45	0.05	com	[36, 37]
	CYP2C9	Diclofenac	>200^*∗*^			[37]
	CYP2C9	Tolbutamide	61.6	88.6	com	[36]
	CYP2D6	Dextromethorphan	>200^*∗*^			[37]
	CYP2E1	Chlorzoxazone	—	—		[36]
	CYP3A4	Midazolam	>200^*∗*^			[37]
		Testosterone	>200^*∗*^			[37]
		Testosterone	219	141	com	[36]
	CE1	*o*-Nitrophenyl acetate		6.9		[44]
	CE2	*o*-Nitrophenyl acetate		2.5		[44]
		Irinotecan		0.07		[44]

Cryptotanshinone	CYP1A1	7-Ethoxyresorufin		2.2^*∗*^		[39]
	CYP1A2	7-Ethoxyresorufin		0.56^*∗*^		[39]
	CYP1A2	Phenacetin	0.68^*∗*^		com	[38]
		Phenacetin	0.45–1.88	0.27	com	[36, 37]
	CYP2C9	Diclofenac	8		mixed	[37]
		Tolbutamide	22.9	9.90	com	[36]
	CYP2D6	Dextromethorphan	68		mixed	[37]
	CYP2E1	Chlorzoxazone	10.87	ND	com	[36]
	CYP3A4	Midazolam	>200^*∗*^			[37]
		Testosterone	>200^*∗*^			[37]
		Testosterone	120	59.9	com	[36]
	CE1	*o*-Nitrophenyl acetate		0.54		[44]
	CE2	*o*-Nitrophenyl acetate		0.14		[44]
		Irinotecan		0.29		[44]

Dihydrotanshinone I	CYP1A2	Phenacetin	0.53	0.02	com	[36]
	CYP2C9	Tolbutamide	1.92	3.14	com	[36]
	CYP2E1	Chlorzoxazone	—	ND	un	[36]
	CYP3A4	Testosterone	2.11	2.98	non	[36]
		Midazolam	1.2^*∗*^			[33]
	CE1	*o*-Nitrophenyl acetate		0.40		[44]
	CE2	*o*-Nitrophenyl acetate		0.12		[44]
	CE2	Irinotecan		1.83		[44]

Miltirone	CE1	*o*-Nitrophenyl acetate		2.53		[44]
	CE2	*o*-Nitrophenyl acetate		0.04		[44]
	CE2	Irinotecan		0.08		[44]

Tanshinone IIA sulfonate	CYP2A6	Coumarin	>100^*∗*^			[31]
	CYP2C9	Tolbutamide	>100^*∗*^			
	CYP2C19	s-mephenytoin	>100^*∗*^			
	CYP2D6,	Metoprolol	>100^*∗*^			
	CYP2E1	Chlorzoxazone	>100^*∗*^			
	CYP3A4	Midazolam	3.2		com	
	CE1	*o*-Nitrophenyl acetate		>100		[44]
	CE2	*o*-Nitrophenyl acetate		3.9		
	CE2	Irinotecan		28.8		

Tanshinol	CYP1A2	Phenacetin	110^*∗*^			[37]
	CYP2C9	Diclofenac	35		com	
	CYP2D6	Dextromethorphan	>200^*∗*^			
	CYP3A4	Midazolam	>200^*∗*^			
		Testosterone	>200^*∗*^			
	OAT1	*p*-Aminohippuric acid		40.4		[54]
				98^*∗*^		[56]
	OAT2	Prostaglandin F_2*α*_		1528^*∗*^		[56]
	OAT3	Estrone sulfate		8.6		[54]
				2803^*∗*^		[56]
	OAT4	Estrone sulfate		4079^*∗*^		[56]

Protocatechuic aldehyde	CYP1A2	Phenacetin	>200^*∗*^			[37]
	CYP2C9	Diclofenac	>200^*∗*^			
	CYP2D6	Dextromethorphan	>200^*∗*^			
	CYP3A4	Midazolam	130^*∗*^			
		Testosterone	160^*∗*^			

Protocatechuic acid	CYP1A2	Phenacetin	>200^*∗*^			[37]
	CYP2C9	Diclofenac	>200^*∗*^			
	CYP2D6	Dextromethorphan	>200^*∗*^			
	CYP3A4	Midazolam	>200^*∗*^			
		Testosterone	>200^*∗*^			

Salvianolic acid B	CYP1A2	Phenacetin	105^*∗*^			[37]
	CYP2C9	Diclofenac	>200^*∗*^			
	CYP2D6	Dextromethorphan	>200^*∗*^			
	CYP3A4	Midazolam	>200^*∗*^			
		Testosterone	>200^*∗*^			
	OAT1	*p*-Aminohippuric acid		22.2		[54]
	OAT3	Estrone sulfate		19.8		

Salvianolic acid A	OAT1	*p*-Aminohippuric acid		5.6		[54]
	OAT3	Estrone sulfate		0.16		

Lithospermic acid	OAT1	*p*-Aminohippuric acid		20.8		[54]
	OAT3	Estrone sulfate		0.59		

Rosmarinic acid	OAT1	*p*-Aminohippuric acid		0.35		[54]
	OAT3	Estrone sulfate		0.55		

Danshen extract^a^	CYP1A2	Phenacetin	190^#^			[64]

Danshen extract^b^	CYP3A4	Testosterone	51^#^		com	[35]

Danshen ethanol extract^c^	CYP1A2	Phenacetin	3.4^#^		com	[34]
	CYP3A4	Testosterone	11.9^#^		com	

Danshen ethanol extract^d^	CYP3A4	Midazolam	8.6^#^			[33]

Salvianolate^e^	CYP1A2,	Phenacetin	>100^*∗*#^			[32]
	CYP2A6	Coumarin	>100^*∗*#^			
	CYP2C9	Tolbutamide	>100^*∗*#^			
	CYP2C19	s-mephenytoin	>100^*∗*#^			
	CYP2D6,	Metoprolol	>100^*∗*#^			
	CYP2E1	Chlorzoxazone	>100^*∗*#^			
	CYP3A4	Midazolam	2.27^#^	3.58^#^	non	

com, competitive; non, noncompetitive; un, uncompetitive.

*∗*, IC50 (*μ*M).

#, *μ*g/mL.

^a^Containing tanshinol 3.1, salvianolic acid B 37.3, rosmarinic acid 1.8 and protocatechuic aldehyde 0.16 mg/g, and dihydrotanshinone I 12.7, cryptotanshinone 34.6, tanshinone I 10.4, and tanshinone IIA 22.6 *μ*g/g.

^b^Containing tanshinol 31, salvianolic acid B 41, rosmarinic acid 6.1, and protocatechuic aldehyde 2.8 mg/g, dihydrotanshinone I 98, cryptotanshinone 70, tanshinone I 32, and tanshinone IIA 21 *μ*g/g.

^c^Containing tanshinol 2.68, salvianolic acid B 209, rosmarinic acid 14.9, and protocatechuic aldehyde 0.64, dihydrotanshinone I 9.3, cryptotanshinone 36.8, tanshinone I 17.9, and tanshinone IIA 118 mg/g.

^d^Containing tanshinone IIA 106.2, cryptotanshinone 88.0, tanshinone I 53.1, and dihydrotanshinone I 13.5 mg/g.

^e^Containing salvianolic acid B (≥85%), rosmarinic acid (≥10.1%), and lithospermic acid (≥1.9%).

**Table 2:** Clinical trials on pharmacokinetic Danshen-drug interactions.

Dose regimen				Effect and ratio (treatment/control)	Reference
Danshen	Daily dose (mg/day)		Victim drug		
Four Danshen extract tablets (each tablet contained an extract of 1 g Danshen), p.o., three times daily for 14 days (days 2–15)	Cryptotanshinone, tanshinone, tanshinone IIA, tanshinol, protocatechuic acid, salvianolic acid B	3.0 6.0 4.2 19.2 2.4 156	A single oral dose of theophylline at 100 mg on days 1 and 15.	No effect	[65]
Sodium tanshinone IIA sulfonate injections, i.v., 60 mg/day for 13 days			Baseline and a single oral dose of caffeine at 100 mg on day 13 after injection of placebo or sodium tanshinone IIA sulfonate.	CYP1A2 activity (ratio of paraxanthine to caffeine at 6 h in plasma) ratio 1.41; caffeine AUC ratio 0.87, paraxanthine AUC ratio 1.17	[66]
Four Danshen extract tablets (each tablet contained an extract of 1 g Danshen), p.o., three times daily for 14 days (days 2–15)	Cryptotanshinone tanshinone I, tanshinone IIA, protocatechuic aldehyde, tanshinol, salvianolic acid B	3.12 6.0 4.44 8.04 20.4 162	A single oral dose of midazolam at 15 mg on days 1 and 16.	Midazolam AUC ratio 0.74, *C*_max_ ratio 0.69, CL/F ratio 1.35. No effect on *C*_max_ and AUC ratios of midazolam to 1-hydroxymidazolam	[67]
Ethanol extract of Danshen at 1 g, p.o., a single dose	Tanshinone IIA, cryptotanshinone, tanshinone I, dihydrotanshinone I	106.2 88.0 53.1 13.5	Baseline and a single oral dose of midazolam at 15 mg 0.5 h after administration of 1 g Danshen extract.	Midazolam *C*_max_ ratio 1.87; 1-hydroxymidazolam *C*_max_ ratio 1.68	[33]
Ethanol extract of Danshen at 1 g, p.o., three times a day for 10 days (days 2–11)	Tanshinone IIA, cryptotanshinone, tanshinone I, dihydrotanshinone I	318.6 264.0 159.3 40.5	A single oral dose of midazolam at 15 mg of on days 1 and 12 (0.5 h after administration of 1 g Danshen extract).	Midazolam AUC_0–12 h_ ratio 0.20, *C*_max_ ratio 0.34, and *t*_1/2_ ratio 0.56, CL/F ratio 6.0; 1-hydroxymidazolam AUC_0–12 h_ ratio 0.55; AUC_1-hydroxymidazolam_/AUC_midazolam_ ratio 3.0	
T89 (225 mg, twice daily, p.o.) and warfarin were given simultaneously for one-week after reaching warfarin steady state			Warfarin dose to maintain international normalized ratio at 1.3–1.9 was selected.	T89 has no effect on the steady‐state PK of warfarin	[68]
Ethanol extract of Danshen at 1 g, p.o., three times a day for 10 days (days 2–11)	Tanshinone IIA, cryptotanshinone, tanshinone I	318.6 264.0 159.3	A single oral dose of fexofenadine at 60 mg on days 1 and 12.	*C*_max_ ratio 0.65, AUC_0–24 h_ ratio 0.55, CL/F ratio 2.11. Induction of intestinal P-glycoprotein	[62]

p.o., oral.

i.v., intravenous.
